# Effects of heat treatment and ultraviolet radiation on physicochemical, microbiological, and bioactive properties of shalgam juice

**DOI:** 10.1002/fsn3.4445

**Published:** 2024-09-10

**Authors:** Kubra Feyza Erol, Gozde Kutlu, Mehmet Baslar, Fatih Tornuk

**Affiliations:** ^1^ Department of Nutrition and Dietetics University of Health Sciences, Hamidiye Faculty of Health Sciences Istanbul Türkiye; ^2^ Department of Gastronomy and Culinary Arts Ankara Medipol University, Faculty of Fine Arts, Design and Architecture Ankara Türkiye; ^3^ Department of Gastronomy and Culinary Arts Istanbul Arel University, Faculty of Fine Arts Istanbul Türkiye; ^4^ Department of Nutrition and Dietetics Sivas Cumhuriyet University, Faculty of Health Sciences Sivas Türkiye

**Keywords:** in vitro bioaccessibility, microbiological properties, physicochemical properties, sensory properties, shalgam juice

## Abstract

Herein, we aimed to investigate the physicochemical, bioactive, microbial, and sensory properties of 5 different ultraviolet (UV) treatment conditions, varying in flow rate (1.5–2.5–3.5 L/min), temperature (5–25°C), and UV‐intensities (5.1–10.1 mW/cm^2^), along with heat treatment (HT, 72°C for 20 s), and untreated samples (C) over a storage period of 150 days. At the beginning of storage, the samples exhibited a dry matter content ranging from 2.15% to 2.38%, pH levels ranging from 3.46 to 3.53, and total acidity between 6.35 and 6.51 mg/L. L* values were recorded between 33.09 and 33.50, while *ΔC* values ranged from 9.13 to 9.24. However, by the end of storage, these values had changed to 2.28–2.43% for dry matter, 3.47–3.49 for pH, 6.22–6.35 mg/L for acidity, 34.94–35.57 for L* values, and 6.95–7.01 for *ΔC* values. Throughout storage, total mesophilic aerobic bacteria (TMAB), lactic acid bacteria (LAB), and yeast and molds were measured at the highest levels in the C samples when compared to HT and UV‐treated samples. At the end of storage, compared to the initial values, TMAB levels in UV‐treated samples decreased from 3.29–4.80 log cfu/mL to 3.13–3.92 log cfu/mL. On the other hand, compared to the initial values (3.29–4.01 log cfu/mL), LAB levels decreased by 1.93–2.42 log cfu/mL by the end of storage. Initially, in UV‐treated samples, the TPC (total phenolic content) ranged from 398.15 to 403.86 mg GAE/g, DPPH antioxidant activity ranged from 811.52 to 834.89 mg TE/L, and TAC (total anthocyanin content) ranged from 5.58 to 5.74 mg/L. By the end of storage, an increase was observed in all bioactive properties analyzed. Furthermore, UV treatment positively impacted the bioaccessibility of bioactive compounds compared to the HT‐treated sample. Overall, this study confirms that UV‐C technology can be used as an alternative method for extending the shelf life of shalgam juice while preserving its sensory and bioactive attributes.

## Graphical abstract

In this study, the effect of UV‐C as an alternative to heat treatment was applied to shalgam juice processing. UV application provided better bioactive properties than heat treatment. The microbiological properties of shalgam were held within the limits of Turkish standards.

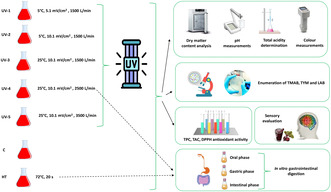

## 1 INTRODUCTION

Shalgam juice is a low‐acidic, lactic acid‐fermented, non‐alcoholic, traditional Turkish drink designed for fresh consumption. According to the Turkish Standards, shalgam juice (fermented turnip juice, şalgam) is defined as a product prepared by combining potable water (TS 266), bulgur flour, edible salt (TS 933), and sourdough. The mixture undergoes spontaneous lactic acid fermentation following the addition of purple carrot (*Daucus carota*), şalgam (*Brassica rapa*), and powdered hot pepper (TS 241) into the extracted liquid. This mixture is then subjected to another round of lactic acid fermentation (Ekinci et al., 2016). Lactic acid, apart from giving shalgam juice its characteristic sour taste, offers various digestive benefits, imparts a cooling sensation, helps regulate the pH of the digestive system, and aids in the absorption of certain minerals by the body (Aladeboyeje & Şanli, 2021; Gok, 2021). Shalgam juice is widely consumed, especially in most Turkish regions, such as Hatay, Mersin, Osmaniye, Adana, and Kahramanmaraş, where it is both homemade and commercially produced. Recently, its export to European countries have increased, making it a growing sector in Türkiye. As consumption rises, larger production facilities are replacing smaller ones, necessitating research and development efforts to improve production techniques, standardize processes, and extend shelf life (Akman et al., 2021; Tangüler et al., 2020; Tanriseven et al., 2018).

UV‐irradiation is a non‐toxic food processing technique that is widely known for its antimicrobial effects. UV‐C light (short‐wave ultraviolet light, 200–280 nm) is particularly effective at damaging the DNA of pathogenic microorganisms. Its use offers a potential reduction in reliance on chemical disinfectants in the food industry. UV‐C light has been successfully applied in various areas of food processing, including liquid foods like juices, alcoholic beverages, and milk, as well as in the preservation of ready‐to‐eat meat products and fresh foods. The United States Food and Drug Administration (FDA) has officially approved UV‐irradiation as a safe method for food sanitation (Antonio‐Gutiérrez et al., 2019; Dogan et al., 2021; Fundo et al., 2021).

The industrial manufacturing of shalgam juice faces challenges related to standardization of starter cultures, overbalance of batch production, and the need for multiple preservatives to extend the shelf life. Heat treatment and antimicrobial agents are commonly used together; however they can negatively impact the drink's sensory quality. Sediments at the bottle bottom also negatively affect visual appeal and consumer preference (Ates et al., 2021; Kabak & Dobson, 2011; Ulucan et al., 2022). To meet consumer demand for high‐quality natural shalgam juice, non‐thermal food processing methods like high hydrostatic pressure (Ates et al., 2021), pulsed ultraviolet light (Karaoglan et al., 2017, 2019), ultrasonication (Irkilmez, 2017), and ultrasound processes (Ulucan et al., 2022) have been tested. However, to the best of our knowledge, no previous research has focused on using UV‐C irradiation to extend the shelf life of shalgam juice. Therefore, this study aimed to determine the effect of different UV‐C treatment conditions on microbial, physico‐chemical, bioactive, and sensory properties as well as the in vitro bioaccessibility of bioactive compounds in shalgam juice during a 150‐day storage period.

## 2 MATERIALS AND METHODS

### 2.1 Materials

Fresh (spontaneously fermented but not pasteurized) shalgam juice (salt and ash contents: 1.79 ± 0.08% and 1.43 ± 0.10%, respectively) was obtained from Sena Food Ind.Trade.Co.Ltd. (Adana, Türkiye), which is commercially known as Hacının Şalgamı.

### 2.2 Ultraviolet (UV) and heat treatment (HT)

**FIGURE 1:**
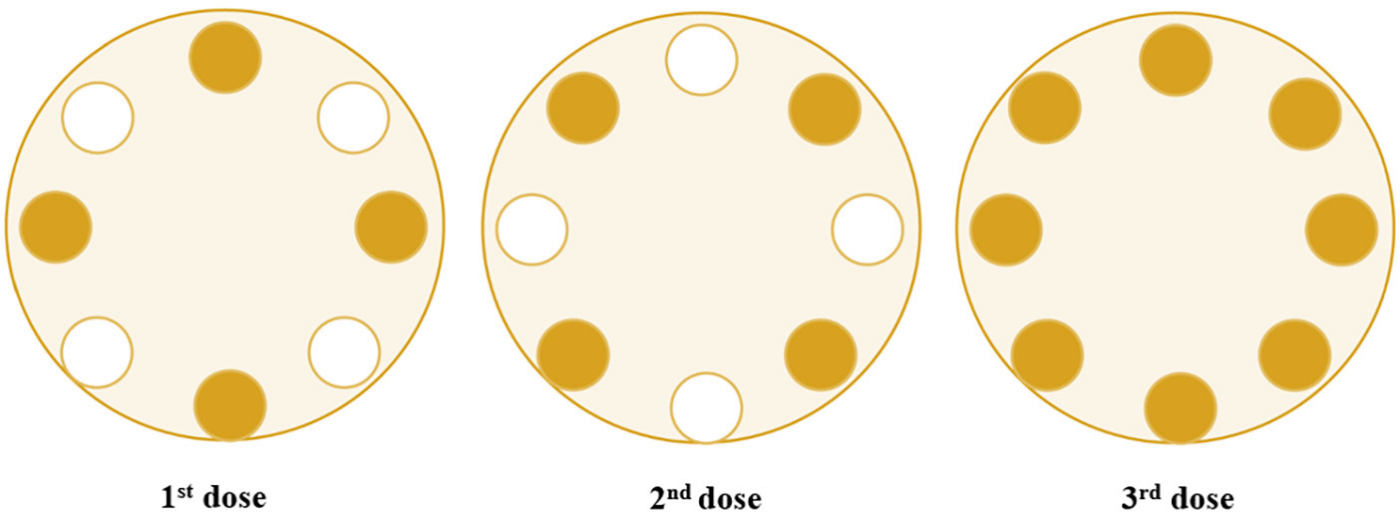
The design of ultraviolet (UV) lamps for the first dose of 5.1 mW/cm^2^, the second dose of 5.1 mW/cm^2^, and the third dose of 10.1 mW/cm^2^ (Dogan et al., 2021).

Shalgam juice samples were divided into 7 lots and subjected to UV and HT as listed in Table 1. Five different UV doses were applied to the samples using a UV device (CiderSure 3500, USA), considering varying flow rates (1.5, 2.5, and 3.5 L/min), UV doses (Figure 1) (Dogan et al., 2021), and temperatures (5 and 25°C). As a control (C), one sample was left untreated and not subjected to any pasteurization process. After different treatments, the juices were aseptically filled in 100‐mL glass bottles, sealed, and stored at +4°C for 150 days.

**TABLE 1:** Different processing conditions applied to shalgam juices.

Operation codes	Temperature (°C)	UV lamp series	UV dose (mV/cm^2^)	Flow rate (L/min)	Time (s)
UV‐1	5	1	5.1	1500	‐
UV‐2	5	1 + 2	10.1	1500	‐
UV‐3	25	1 + 2	10.1	1500	‐
UV‐4	25	1 + 2	10.1	2500	‐
UV‐5	25	1 + 2	10.1	3500	‐
C	‐	‐	‐	‐	‐
HT	72	‐	‐	‐	20

Abbreviations: C, untreated sample; HT, heat treatment; UV, ultraviolet treatment.

### 2.3 Physico‐chemical analyses

The total dry matter of the samples was assessed gravimetrically. Samples were dried at 105°C for 2.5 h in a Memmert UF‐110 drying oven, following Cemeroğlu's (2013) protocol. For pH measurement (Burun et al., 2024), the sample was placed in a beaker at 22 ± 2°C, with the pH meter electrode directly immersed (Kutlu et al., 2024). Percent acidity as lactic acid was determined according to the method of Cemeroğlu (2013), and the lactic acid content was calculated using the following formula:

(1) $$\text{Titration acidity}=\frac{\left(V*F*E*100\right)}{M}$$

where *V* was the volume of 0.1 N NaOH solution used (ml), *F* was the factor of the base solution, *E* was the equivalent weight of acid (g), and *M* was the sample volume (mL) titrated.

The color measurements of shalgam samples were performed using a calibrated colorimeter (CR‐400, Konica Minolta Sensing, Inc., Japan) with a white reference plate, following the manufacturer's instructions. For this purpose, approximately 5 mL of the shalgam sample was placed in a clean, clear glass measurement container and was gently stirred to achieve uniform consistency. At least three measurements were taken to determine the L* (color brightness; lightness (white, L* = 100), darkness (black, L* = 0)), a* (red (+a*)‐green (−a*)), and b* (yellow (+b*)‐blue (−b*)) values. The measurement results were averaged to obtain representative color values for each sample. *ΔC* values were calculated from the obtained a* and b* values using the following equation (Yavuz et al., 2022):

(2) $$\Delta C=\sqrt{{\left(a^{*}\right)}^{2}+{\left(b^{*}\right)}^{2}}$$

### 2.4 Microbiological analysis

The microbiological properties of the shalgam samples were assessed by evaluating total mesophilic aerobic bacteria (TMAB), total yeasts and molds (TYM), total coliform bacteria (TCB), and lactic acid bacteria (LAB) counts. For this purpose, 25 mL of the sample was mixed with 225 mL of sterile peptone water, and subsequent dilutions were prepared. Then, suitable dilutions of the samples were spread onto PCA, DRBC, VRB, and MRS agar plates, and the plates were incubated at 30°C for 48 h, at 25°C for 5 days, at 30°C for 48 h and 37°C for 72 h (aerobically and anaerobically), respectively (Bagdat, Akman, et al., 2024; Bagdat, Kutlu, & Tornuk, 2024).

### 2.5 Determination of bioactive properties

Several bioactive attributes of the shalgam samples were analyzed, including total phenolic content (TPC), DPPH radical scavenging activity, and total anthocyanin content (TAC), using a UV/VIS spectrophotometer (Shimadzu UV‐1800, Japan). Additionally, changes in the in vitro bioaccessibility of these bioactive properties was also studied.

### 2.6 Extraction procedure

Five mililiters of shalgam juice were incorporated with 80% aqueous methanol (25 mL) and homogenized at room temperature for 2.5 h using a shaker (Hei‐MIX Shakers and Mixers from Heidolph, Germany). After filtering the solutions through filter paper, they were centrifuged at 4100 rpm at 25°C for 10 min using a Hettich Universal 320 R centrifuge (Germany). The supernatant was then collected and used for further experiments.

### 2.7 TPC

The Folin–Ciocalteu procedure, based on the method originally described by Kutlu (2024), was used to determine the TPC of the samples (0.5 mL) at 760 nm by applying the equation (y (mg GAE (gallic acid equivalent)/g)) = (x‐0.0821/0.0111 × dilution factor).

### 2.8 DPPH

The DPPH radical scavenging activities of the shalgam juice samples (0.1 mL) were determined by measuring the absorbance values at 517 nm spectrophotometrically, following the procedure reported by Yasar et al. (2022). The results were presented as mg TE (trolox equivalent)/L using the equation (y = (x‐0.0542)/1.5902).

### 2.9 TAC

TAC analysis was conducted by the spectrophotometric measurements at 510 nm and 700 nm using a pH differential method as previously suggested by Sánchez‐Moreno (2002). The amount of cyanide 3‐glycoside presented in shalgam juice was given as mg/L and calculated based on the following equation:

(3) $$\begin{array}{c} \mathrm{TAC}={\left({\text{Absorbance}}_{0}-{\text{Absorbance}}_{700}\right)}_{\mathrm{pH}:1.0}\\ \;-{\left({\text{Absorbance}}_{0}–{\text{Absorbance}}_{510}\right)}_{\mathrm{pH}:4.5} \end{array}$$

### 2.10 The simulated in vitro digestion

The in vitro bioaccessibility assay of UV‐ or HT‐treated shalgam juice samples stored for 150 days was carried out based on the 3‐step method used by Minekus et al. (2014). The effects of in vitro digestion conditions on TFC, TAC, and DPPH antiradical activities of post‐digestion samples was also determined. As well, bioaccessibility index (BI, %) values were calculated using the following equation (Equation 4). In this equation, IN refers to the fraction that is dialyzable during intestinal digestion, representing the portion inside the dialysis tube while OUT represents the fraction outside the dialysis tube during intestinal digestion, described as the non‐dialyzable portion.

(4) $$\mathrm{BI}\;\left(\%\right)=\frac{\text{Amount of subs}\text{tan}\text{ce in the}\;\text{IN phase}}{\text{Amount of subs}\text{tan}\text{ce in the}\;\text{IN+OUT}\;\text{phases}\;}\times 100$$

### 2.11 Sensory analysis

The sensory properties of the samples were analyzed using a 9‐point hedonic scale (ranging from 1 to 9) with 10 semi‐trained panelists. The samples were assessed by the panelists in terms of appearance, taste, odor, aroma, color, turbidity, consistency, and overall acceptability. To obtain unbiased results, the panelists were advised to refrain from smoking, eating, or consuming coffee for at least 1 h prior to the test. The sensory evaluation was carried out in a controlled setting to ensure consistent conditions.

### 2.12 Statistical evaluation

This study was conducted with 3 parallels and 2 replicates. The collected data were treated with a one‐factor ANOVA to identify the significant differences at a 95% confidence level through Duncan's multiple range test. Statistical analysis was performed using SPSS 20.0 software for Windows.

## 3 RESULTS AND DISCUSSION

### 3.1 Physicochemical properties

Table 2 illustrates the changes in dry matter content of shalgam juice samples during the storage. The dry matter values ranged from 2.15% to 2.39%, depending on the treatment and storage duration. However, no significant difference in dry matter content was observed during storage (*p* > 0.05). Similarly, Özer and Çoksöyler (2015) reported that 26 shalgam juice samples had dry matter contents ranging from 2.0 to 3.2%.

**TABLE 2:** Changes in dry matter, pH, and total acidity of shalgam beverage samples.

Parameters	Operation codes	Storage days						
		0	15	30	60	90	120	150
Dry matter (%)	UV‐1	2.34 ± 0.02^Aa^	2.32 ± 0.12^Aa^	2.22 ± 0.11^Aa^	2.28 ± 0.01^Aa^	2.25 ± 0.02^Aa^	2.27 ± 0.03^Aa^	2.43 ± 0.28^Aa^
	UV‐2	2.38 ± 0.07^Aa^	2.31 ± 0.07^Aa^	2.14 ± 0.11^Aa^	2.32 ± 0.01^Aa^	2.18 ± 0.04^Aa^	2.29 ± 0.06^Aa^	2.28 ± 0.11^Aa^
	UV‐3	2.15 ± 0.19^Aa^	2.23 ± 0.12^Aa^	2.18 ± 0.07^Aa^	2.34 ± 0.03^Aa^	2.17 ± 0.01^Aa^	2.16 ± 0.17^Aa^	2.37 ± 0.20^Aa^
	UV‐4	2.33 ± 0.00^Aa^	2.28 ± 0.15^Aa^	2.90 ± 0.01^Aa^	2.32 ± 0.05^Aa^	2.19 ± 0.12^Aa^	2.24 ± 0.05^Aa^	2.38 ± 0.15^Aa^
	UV‐5	2.32 ± 0.12^Aa^	2.25 ± 0.01^Aa^	2.29 ± 0.03^Aa^	2.35 ± 0.06^Aa^	2.17 ± 0.04^Aa^	2.39 ± 0.02^Aa^	2.34 ± 0.21^Aa^
	C	2.27 ± 0.00^Aa^	2.09 ± 0.03^Aa^	2.20 ± 0.01^Aa^	2.23 ± 0.04^Aa^	2.18 ± 0.00^Aa^	2.16 ± 0.16^Aa^	2.38 ± 0.19^Aa^
	HT	2.37 ± 0.24^Aa^	2.25 ± 0.06^Aa^	2.20 ± 0.01^Aa^	2.29 ± 0.01^Aa^	2.30 ± 0.08^Aa^	2.16 ± 0.03^Aa^	2.40 ± 0.18^Aa^
pH	UV‐1	3.46 ± 0.01^Ade^	3.46 ± 0.05^Ade^	3.35 ± 0.00^Ac^	3.43 ± 0.01^Ad^	3.20 ± 0.01^Ab^	3.12 ± 0.00^Aa^	3.49 ± 0.06^Ae^
	UV‐2	3.46 ± 0.02^ABde^	3.49 ± 0.04^Ae^	3.36 ± 0.01^ABc^	3.45 ± 0.01^Ad^	3.23 ± 0.01^Ab^	3.13 ± 0.03^Aa^	3.47 ± 0.05^Ae^
	UV‐3	3.47 ± 0.0^ABCd^	3.48 ± 0.04^Ad^	3.39 ± 0.02^Cc^	3.50 ± 0.10^Ad^	3.22 ± 0.04^Ab^	3.15 ± 0.04^Aa^	3.48 ± 0.06^Ad^
	UV‐4	3.49 ± 0.02^Cd^	3.45 ± 0.04^Ad^	3.36 ± 0.01^ABc^	3.50 ± 0.08^Ad^	3.20 ± 0.00^Ab^	3.13 ± 0.04^Aa^	3.49 ± 0.07^Ad^
	UV‐5	3.49 ± 0.01^BCd^	3.47 ± 0.03^Ad^	3.35 ± 0.01^Ac^	3.49 ± 0.09^Ad^	3.23 ± 0.05^Ab^	3.14 ± 0.01^Aa^	3.49 ± 0.06^Ad^
	C	3.53 ± 0.01^Df^	3.47 ± 0.07^Ade^	3.38 ± 0.03^BCc^	3.44 ± 0.03^Acd^	3.21 ± 0.01^Ab^	3.08 ± 0.11^Aa^	3.48 ± 0.06^Ade^
	HT	3.49 ± 0.03^Cc^	3.47 ± 0.05^Ade^	3.37 ± 0.02^ABCc^	3.43 ± 0.01^Ad^	3.20 ± 0.01^Ab^	3.13 ± 0.04^Aa^	3.49 ± 0.07^Ae^
Total acidity as lactic acid (mg/L)	UV‐1	6.44 ± 0.06^ABc^	6.17 ± 0.06^Ab^	5.90 ± 0.06^Aa^	6.40 ± 0.10^Bc^	6.67 ± 0.03^Ad^	6.80 ± 0.06^ABd^	6.22 ± 0.04^Ab^
	UV‐2	6.51 ± 0.05^Bc^	6.15 ± 0.02^Aa^	6.01 ± 0.08^ABa^	6.31 ± 0.10^ABb^	6.55 ± 0.02^Ac^	6.88 ± 0.00^Bd^	6.31 ± 0.03^Ab^
	UV‐3	6.51 ± 0.02^Bd^	6.10 ± 0.02^Ab^	5.90 ± 0.06 ^Aa^	6.22 ± 0.06^ABc^	6.67 ± 0.03^Ae^	6.71 ± 0.03^Ae^	6.26 ± 0.05^Ac^
	UV‐4	6.42 ± 0.02^ABc^	6.22 ± 0.10^Ab^	6.01 ± 0.02^ABa^	6.26 ± 0.06^ABb^	6.58 ± 0.03^Ad^	6.78 ± 0.02^Abe^	6.26 ± 0.03^Ab^
	UV‐5	6.35 ± 0.03^Ac^	6.15 ± 0.11^Ab^	5.95 ± 0.03^Aa^	6.31 ± 0.03^Abc^	6.55 ± 0.08^Ad^	6.82 ± 0.05^Abe^	6.31 ± 0.04^Abc^
	C	6.35 ± 0.06^Ab^	6.31 ± 0.10^Aab^	6.15 ± 0.05^Ba^	6.42 ± 0.05^ABb^	6.62 ± 0.06^Ac^	7.16 ± 0.06^Cd^	6.35 ± 0.05^Ab^
	HT	6.42 ± 0.08^ABc^	6.24 ± 0.08^Ab^	5.97 ± 0.05^Aa^	6.33 ± 0.08^ABbc^	6.64 ± 0.05^Ad^	6.80 ± 0.03^Abe^	6.35 ± 0.02^Abc^

*Note*: Lowercase letters denote significant statistical differences within rows via the ANOVA Duncan test (*p* < .05); identical letters imply no statistical difference between samples (*p* ≥ .05). Uppercase letters indicate significant differences within columns as per the ANOVA Duncan test.

The pH and acidity of shalgam juice, influenced by lactic acid fermentation, impact its organoleptic qualities, with lactic acid being the predominant organic acid resulting from this process (Ekinci et al., 2016; Tanriseven et al., 2018). The pH values of shalgam juices were varied from 3.08 to 3.53 (Table 2). According to Turkish standards (TSE 11149, 2003), the pH value of shalgam should be between 3.3 and 3.8. The data indicated that the variation in pH values during storage was slight and insignificant (*p* > .05). Samples stored for 90 and 120 days had slightly lower pH values, but after 150 days, pH reached the desired levels in terms of TSE‐standards. The increase in pH values and the decrease in acidity during storage could be attributed to the oxidative degradation of lactic acid by yeasts (Kırlangıç et al., 2021). Likewise, Turker et al. (2004) observed minor pH changes in shalgam juice over 90 days, with values ranging from 3.54 to 3.76.

The fermentation process of shalgam is predominantly carried out by homofermentative and heterofermentative LAB. Lactic acid fermentation not only helps to preserve the fermented food but also contributes to its taste and aroma (Tanguler & Erten, 2012). As seen in Table 2, the total acidity values of the shalgam juices varied from 5.90 to 6.80 mg/L, as consistent with the findings of Deryaoğlu (1990). Acidity decreased in all samples by the day 30, increased from 30 to 120 days, and then slightly decreased on the day 150. At the end of 150^th^ day, the total acidity of the C‐sample remained unchanged, while all other samples decreased slightly as compared to the initial values. The acidity of UV‐C‐treated samples remained lower than that of C and HT after 150 days, but UV‐5 and HT showed no significant difference (*p* > .05). At the end of storage, UV‐C‐treated samples exhibited similar acidity levels, ranging from 6.22 to 6.31 mg/L. No significant difference was observed (*p* > .05) between C/UV‐1/UV‐2/UV‐4 and UV‐5/HT at 150^th^ day. Per TSE 11149 (2003), shalgam juice should have ≥ 6.0 g/L titratable acidity as the lactic acid equivalent. UV‐3, UV‐5, and HT‐samples fell below this standard on the day 30. However, acidity levels remained within the specified limits within the extending storage period

The intense purplish‐red color of shalgam comes from black carrot, which also adds significant amounts of anthocyanins. Shalgam has a large popularity due to its distinctive color, spicy taste, and aroma. Therefore, any processing methods applied to shalgam must preserve its physical attributes, especially color and sensory qualities, as these can be easily compromised by processing parameters (Ates et al., 2021). Table 3 presents *ΔC* values ranging from 6.43 to 9.24 and L* values ranging from 25.19 to 35.66 for shalgam juice. Both L* and *ΔC* values were significantly affected from storage time and treatment (*p* < .05). The *ΔC* value is a measure that represents the brilliance or purity of color, indicating the level of color saturation or intensity. The highest and lowest *ΔC* values were observed on the days 0 and 90, respectively. However, *ΔC* values increased slightly after this period. Color change in shalgam may be linked to the activity of starter cultures during storage (Kırlangıç et al., 2021). Until the 15^th^ day, the L* values increased compared to the initial values, while between a decrease was observed up to the 120^th^ day. On the day 150, the L* values increased again. At the end of the 150‐day storage period, the increase in L* values was lower in UV‐1, UV‐2, and HT samples compared to the control samples, whereas it was higher in UV‐3, UV‐4, and UV‐5 samples. According to the study conducted by Mansor et al. (2017), the decrease in L* value was attributed to suspended particle precipitation in juice. Tangüler et al. (2014) found that higher L* values were associated with reduced color intensity in shalgam beverage. Anthocyanins, being the most plentiful group of pigments, are responsible for purple, blue, and red colors (Ates et al., 2021). Color changes may also be attributed to several factors, such as extraction, oxidation, isomerization, and enzymatic co‐oxidation of anthocyanins (Tanriseven et al., 2020). Maintaining the color and sensory qualities of shalgam juice throughout its shelf life involves understanding and controlling these parameters. All these factors play a critical role in the stability and appearance of anthocyanin pigments. By optimizing processing parameters and storage conditions, it is possible to preserve the distinctive purplish‐red color that defines the characteristic appearance of shalgam juice and to maintain its popularity.

**TABLE 3:** Changes in L* and *ΔC* values of shalgam juice samples.

Parameters	Operation codes	Storage days						
		0	15	30	60	90	120	150
L* value	UV‐1	33.49 ± 0.03^Bb^	33.98 ± 0.66^Ab^	29.84 ± 0.27^Ba^	29.86 ± 0.67^Ba^	30.75 ± 1.16^Ba^	31.79 ± 0.29^Ba^	35.41 ± 0.33^Ab^
	UV‐2	33.50 ± 0.04^Bb^	35.66 ± 0.27^Db^	31.06 ± 0.40^Ba^	29.74 ± 0.66^Ba^	31.30 ± 0.87^Ba^	31.56 ± 0.54^Ba^	35.10 ± 0.47^Ab^
	UV‐3	33.18 ± 0.01^Ab^	35.26 ± 0.13^CDb^	31.08 ± 0.^B^	29.54 ± 0.19^Ba^	25.19 ± 0.67^Aa^	31.37 ± 0.40^Aa^	35.57 ± 0.52^Bb^
	UV‐4	33.15 ± 0.01^Abc^	34.74 ± 0.24^BCc^	29.84 ± 0.58^Ba^	29.81 ± 0.07^Bab^	29.97 ± 0.30^Ba^	31.51 ± 0.37^Ba^	35.43 ± 0.22^Ab^
	UV‐5	33.09 ± 0.29^Ab^	35.32 ± 0.49^Db^	29.23 ± 0.23^Aa^	29.46 ± 0.20^Aa^	30.02 ± 0.19^Ba^	31.45 ± 0.42^Aa^	35.45 ± 0.01^Ab^
	C	33.26 ± 0.26^Abc^	34.74 ± 0.57^BCc^	29.84 ± 0.39^Ba^	30.01 ± 0.21^Bab^	29.96 ± 0.32^Ba^	31.39 ± 0.71^Aa^	35.53 ± 0.14^Bc^
	HT	33.15 ± 0.05^Abc^	34.30 ± 0.16^ABc^	29.04 ± 0.60^Aa^	30.06 ± 0.45^Bab^	29.44 ± 1.16^Ba^	31.51 ± 0.21^Ba^	34.94 ± 0.64^Ac^
ΔC* values	UV‐1	9.14 ± 0.01^Ac^	8.78 ± 0.51^Ac^	7.98 ± 0.53^Ab^	9.19 ± 0.12^Cc^	6.71 ± 0.59^Aa^	7.08 ± 0.36^Aa^	6.95 ± 0.02^ABa^
	UV‐2	9.13 ± 0.01^Ac^	8.80 ± 0.46^Ac^	7.97 ± 0.41^Ab^	8.96 ± 0.26^ABc^	6.78 ± 0.36^Aa^	6.90 ± 0.31^Aa^	6.98 ± 0.06^ABCa^
	UV‐3	9.24 ± 0.00^Bd^	8.86 ± 0.54^Ac^	8.13 ± 0.31^ABb^	8.84 ± 0.03^Ac^	6.88 ± 0.32^Aa^	6.95 ± 0.40^Aa^	6.92 ± 0.02^Aa^
	UV‐4	9.23 ± 0.03^Bd^	8.85 ± 0.50^Ac^	8.27 ± 0.24^ABCb^	8.92 ± 0.09^ABcd^	6.64 ± 0.35^Aa^	7.01 ± 0.37^Aa^	7.00 ± 0.04^BCa^
	UV‐5	9.18 ± 0.07^ABe^	8.84 ± 0.55^Ad^	7.99 ± 0.07^Ac^	9.04 ± 0.04^BCde^	6.43 ± 0.24^Aa^	7.06 ± 0.25^Ab^	7.03 ± 0.05^Cb^
	C	9.21 ± 0.09^Bc^	8.90 ± 0.60^Abc^	8.64 ± 0.27^Cb^	9.09 ± 0.13^BCc^	6.68 ± 0.55^Aa^	6.98 ± 0.30^Aa^	6.99 ± 0.06^BCa^
	HT	9.23 ± 0.08^Bc^	8.79 ± 0.54^Ab^	8.54 ± 0.34^BCb^	8.91 ± 0.19^ABbc^	6.97 ± 0.24^Aa^	6.98 ± 0.40^Aa^	7.01 ± 0.06^BCa^

*Note*: Lowercase letters denote significant statistical differences within rows via the ANOVA Duncan test (*p* < .05); identical letters imply no statistical difference between samples (*p* ≥ .05). Uppercase letters indicate significant differences within columns as per the ANOVA Duncan test.

### 3.2 Microbiological properties

Table 4 shows the changes in TMAB counts of shalgam juice samples during the storage, ranging from 1.17 to 7.16 log cfu/mL. The highest count (7.16 log cfu/mL) was observed in C‐samples on the day 30, while the lowest count (1.17 log cfu/mL) was found in HT on the day 60. Except for day 0, HT samples consistently had the lowest counts. As shown in the table, the effect of UV‐C conditions on TMAB levels in the samples varied. It was proposed in the literature that UV‐C photons could be absorbed by forming pyrimidine dimers (cytosine and thymine), which could stop microbial growth by inhibiting DNA transcription and translation (Santhirasegaram et al., 2015). At the end of the storage, UV‐1‐ and HT‐treated samples had lower TMAB counts than the initial, while other samples had higher numbers. Furthermore, as expected, the highest TMAB value was recorded in the control sample throughout the storage. As also seen in Table 4, all of the UV‐C treatments were more effective than C‐samples in TMAB inactivation on the day 0. UV‐C treatment resulted in reductions ranging from 1.07 to 3.54 logs, while HT treatment achieved a maximum reduction of 2.76 log on the day 0. However, starting from the 15^th^ day, HT‐treated samples consistently displayed the lowest TMAB values. The impact of UV‐C radiation on microorganisms can vary depending on various factors such as strain of the target microorganism, composition of the medium or substrate, the initial population of microorganisms, shape of microorganism, the power and duration of UV‐C treatment, distance between the UV‐C light source and the product, as well as the thickness of the product being treated (Abdul Karim Shah et al., 2016).

**TABLE 4:** Microbiological properties of the shalgam beverage samples.

Parameters	Operation codes	Storage days						
		0	15	30	60	90	120	150
TMAB (log cfu/mL)	UV‐1	4.80 ± 1.86^Bc^	5.27 ± 0.3^CDcd^	4.29 ± 1.5^BCbc^	3.32 ± 0.02^Bab^	2.99 ± 0.09^Ba^	4.28 ± 0.26^Dbc^	3.13 ± 0.09^Bab^
	UV‐2	2.84 ± 0.33^Aa^	3.96 ± 1.32^BCc^	2.98 ± 0.27^ABa^	2.75 ± 0.40^Ba^	2.60 ± 0.08^Aa^	3.88 ± 0.01^Cab^	3.40 ± 0.01^Bb^
	UV‐3	2.33 ± 0.08^Aab^	4.83 ± 0.43^BCb^	3.19 ± 0.78^ABa^	3.26 ± 1.04^Bab^	3.24 ± 0.42^Bab^	4.02 ± 0.17^Cab^	3.16 ± 0.02^Bab^
	UV‐4	3.02 ± 0.15^Aa^	4.15 ± 1.47^BCDbc^	4.78 ± 1.44^Cc^	2.90 ± 0.31^Ba^	3.06 ± 0.08^Ba^	3.62 ± 0.11^Bab^	3.49 ± 0.02^Cab^
	UV‐5	3.29 ± 0.06^Aab^	3.69 ± 0.55^Bbc^	4.20 ± 1.27^BCd^	2.63 ± 0.72^Ba^	3.31 ± 0.51^Bab^	3.52 ± 0.09^ABbc^	3.92 ± 0.37^Dbc^
	C	5.87 ± 0.43^Cc^	5.72 ± 0.16^Dc^	7.16 ± 0.67^De^	4.91 ± 0.55^Cb^	4.10 ± 0.37^Ca^	5.65 ± 0.14^Ec^	6.51 ± 0.12^Fd^
	HT	3.11 ± 0.08^Ac^	1.40 ± 1.9^Aab^	2.50 ± 0.01^Abc^	1.17 ± 1.65^Aa^	2.50 ± 0.05^Abc^	3.35 ± 0.07^Ac^	2.55 ± 0.22^Abc^
TYM (log cfu/mL)	UV‐1	4.10 ± 0.07^Fab^	4.76 ± 0.37 ^Db^	3.80 ± 0.53^BCa^	3.11 ± 1.51^Ba^	3.25 ± 0.59^BCa^	3.41 ± 0.77^Ba^	3.50 ± 0.07^Ca^
	UV‐2	2.89 ± 0.15^Ca^	3.81 ± 0.64^Cbc^	3.35 ± 0.29 ^Bab^	3.06 ± 0.70 ^Ba^	2.94 ± 0.18 ^Ba^	3.98 ± 0.00^Dc^	2.89 ± 0.37^Ba^
	UV‐3	2.51 ± 0.40^Ba^	2.84 ± 0.15^Bab^	3.65 ± 0.65^Bb^	2.98 ± 1.60^Bab^	3.29 ± 0.32^BCab^	3.63 ± 0.11^BCDb^	2.61 ± 0.04^Ba^
	UV‐4	3.29 ± 0.31^Dab^	3.94 ± 0.33^Cc^	3.48 ± 0.29^Babc^	3.00 ± 0.96^Ba^	3.24 ± 0.23^BCab^	3.50 ± 0.11^BCabc^	3.69 ± 0.05^Cbc^
	UV‐5	3.64 ± 0.27^Eab^	4.20 ± 0.06^Cc^	3.74 ± 0.21^Bb^	3.42 ± 0.25^Ba^	3.55 ± 0.00^CDab^	3.57 ± 0.01^BCabc^	3.39 ± 0.34^Ca^
	C	5.48 ± 0.02^Gc^	6.34 ± 0.03^Ed^	4.24 ± 0.11^Cb^	3.82 ± 0.48 ^Ba^	3.84 ± 0.05^Da^	3.85 ± 0.09^CDa^	3.73 ± 0.07^Ca^
	HT	<2.00^Aa^	1.59 ± 0.16^Acd^	2.02 ± 0.17^Ad^	0.95 ± 0.05^Abc^	<2.00^Aa^	<2.00^Aa^	0.50 ± 0.01^Aab^
LAB (log cfu/mL)	UV‐1	4.01 ± 0.15^Dcd^	4.06 ± 0.07^Ed^	3.28 ± 0.01^Cbcd^	3.11 ± 0.21^Bbc^	3.42 ± 0.02^Cb^	3.01 ± 0.15^Cab^	2.17 ± 0.21^Ba^
	UV‐2	3.56 ± 0.49^Cb^	2.83 ± 0.10^Cab^	3.18 ± 0.35^Bb^	3.36 ± 0.03^Bb^	2.05 ± 0.05^Ba^	3.04 ± 0.12^Cb^	2.23 ± 0.16^Ba^
	UV‐3	2.99 ± 0.33^Bab^	2.65 ± 0.49^Bab^	3.24 ± 0.26^Cb^	3.16 ± 0.15^Bb^	3.26 ± 0.67^b^	2.64 ± 0.14 ^Cab^	2.24 ± 1.66 ^Ba^
	UV‐4	3.29 ± 0.26^BCb^	3.15 ± 0.52^Cb^	3.21 ± 0.35^BCb^	2.72 ± 0.03^Bb^	2.76 ± 0.08^Bb^	2.11 ± 0.11 ^Ba^	1.93 ± 0.04 ^Ab^
	UV‐5	3.49 ± 0.49^Cc^	3.67 ± 0.04^Dc^	3.38 ± 0.50^Cbc^	3.07 ± 1.15^Bbc^	3.32 ± 0.36^Cbc^	2.80 ± 0.58^Cab^	2.42 ± 0.14^Ba^
	C	6.75 ± 0.25^Ef^	6.41 ± 0.03^Fe^	5.36 ± 0.01^Dc^	5.17 ± 0.21^Cbc^	6.10 ± 0.02^Dd^	5.12 ± 0.15^Db^	3.90 ± 0.21^Ca^
	HT	<2.00^Aa^	<2.0^Aa^	<2^Aa^	<2. ^Aa^	<2.00^Aa^	<2.00^Aa^	<2.00^Aa^

*Note*: Lowercase letters denote significant statistical differences within rows via the ANOVA Duncan test (*p* < .05); identical letters imply no statistical difference between samples (*p* ≥ .05). Uppercase letters indicate significant differences within columns as per the ANOVA Duncan test.

According to the Turkish standards (TSE 11149, 2003) on shalgam juice, TMAB count must be ≤ 5 log cfu/mL. Higher TMAB levels (> 5 log cfu/mL) in samples reflect poor hygiene, sanitation, processing, and storage conditions (Kırlangıç et al., 2021). As seen in Table 4, HT and UV‐C‐treated shalgam juices (except UV‐1 at 15 days) met the Turkish standards, while C samples had greater TMAB numbers during refrigerated storage.

Sources of yeast in shalgam include raw materials (bakers' yeast, sourdough) and production vessels (Tanguler & Erten, 2012). As shown in Table 4, the lowest TYM counts were observed in the control sample, while the highest counts were found in the HT samples. UV C‐treated samples provided varying reduction levels in TYM counts. The results showed that TMAB were more sensitive to UV‐C than TYM. This variation may be due to cellular differences such as DNA structure and pyrimidine bases like thymine (Pala & Toklucu, 2013). Turbidity and the dark color of shalgam may also hinder UV‐C light absorption by certain yeasts. Among the UV‐C‐treated samples, UV‐3 demonstrated the highest inhibitory effectiveness on TYM. The observed increase in TYM counts during the storage may suggest a sign of beginning of spoilage in the shalgam juice (Kırlangıç et al., 2021). Variations in shalgam's physicochemical properties (pH, acidity, turbidity, and phenolic compounds) also likely influenced the survival and growth of microorganisms in shalgam during the storage.

Coliforms are food safety indicators. TCB counts remained < 1 cfu/mL throughout the 5‐month period of storage for all samples, which is in conformity with the shalgam standard (TSE 11149, 2003). Similarly, Ulucan et al. (2022) also reported the absence of coliforms in both control and ultrasound‐treated samples.

Shalgam is a lactic acid fermented product, with LABs originating from raw materials such as bulgur dough and other production ingredients, as well as from the fermentation tanks (Ekinci et al., 2016). Table 4 gives LAB counts ranging from < 2.00 to 6.75 log cfu/mL during storage. On day 0, LAB counts in shalgam juice (excluding HT) ranged from 2.99 to 6.75 log cfu/mL. Similarly, Tanguler and Erten (2012) reported LAB levels of 5.54–7.83 log cfu/mL at the end of fermentation. HT led to lower LAB inactivation, while UV‐treated samples showed a reduced LAB population on the day 0. LAB counts in UV‐treated samples fluctuated during storage, but by the day 150, a decrease ranging from 0.75 to 1.84 logs occurred in all samples. Among the UV‐treated samples, UV‐5 had the highest LAB count (2.42 log cfu/mL) at the 150^th^ day while UV‐4 had the lowest (1.93 log cfu/mL) LAB population. No significant difference was observed between UV‐5/UV‐1/UV‐2/UV‐3 (*p* > .05). HT completely eliminated LAB, whereas UV‐C significantly reduced LAB populations.

### 3.3 Bioactive properties

Phenolic compounds in shalgam beverage contribute to its sourness and provide it antioxidative and antimicrobial attributes. Phenolic levels indicate physical state and quality change risk (haze, browning, and sediment) in fruit based products (Karaoglan et al., 2017). Table 5 displays the TPCs of shalgam drinks stored for 150 days. No specific trend was observed in TPC values throughout the storage. However, UV‐C‐treated samples had significantly higher TPC levels at the en of the storage for 150 days, while C and HT‐samples exhibited lower TPCs as compared to their initial values. The rise in TPC may be linked to the accumulation of polyphenolic compounds as a protective mechanism against UV radiation (Bhat, 2016). Moreover, HT‐treated samples showed the most notable decrease in TPC. Extended HT in juices could also lead to the generation of hydroxymethylfurfural and furfural via Maillard reactions, which are potentially carcinogenic compounds (Hernandez et al., 1997).

**TABLE 5:** Some bioactive properties of the shalgam juice samples.

	Operation codes	Storage days						
		0	15	30	60	90	120	150
TPC (mg GAE/g)	UV‐1	402.06 ± 6.20^ABbc^	398.30 ± 8.30^Ab^	413.69 ± 0.50^ABd^	408.06 ± 2.80^BCcd^	414.01 ± 2.50^Ad^	326.00 ± 2.40^BCa^	471.11 ± 1.50^Ce^
	UV‐2	399.50 ± 4.00^ABb^	404.49 ± 3.00^BCc^	418.20 ± 2.20^BCd^	404.23 ± 5.40^ABCc^	425.33 ± 3.60^Be^	333.00 ± 0.10^DEa^	474.68 ± 1.90^Cf^
	UV‐3	398.15 ± 6.10^Ab^	399.73 ± 1.30^ABbc^	410.77 ± 4.10^Ad^	404.35 ± 3.70^ABCc^	417.15 ± 1.70^Ae^	324.45 ± 3.70^Ba^	481.89 ± 7.50^CDf^
	UV‐4	398.38 ± 5.00^Ab^	398.08 ± 3.00^Ab^	420.38 ± 1.70^Cc^	401.68 ± 1.20^Ab^	425.44 ± 2.90^Bc^	336.45 ± 9.50^Ea^	490.88 ± 11.90^Dd^
	UV‐5	403.86 ± 4.20^ABb^	400.43 ± 3.10^ABb^	420.83 ± 5.90^Cc^	403.71 ± 6.20^ABb^	428.48 ± 6.40^Bc^	356.30 ± 8.50^Fa^	487.35 ± 12.70^Dd^
	C	428.02 ± 0.20^Cd^	406.56 ± 1.90^Cc^	422.93 ± 6.60^Cd^	399.37 ± 2.10^Ab^	424.28 ± 0.60^Bd^	313.15 ± 3.90^Aa^	422.68 ± 7.00^Bd^
	HT	406.19 ± 6.70^Bcd^	399.95 ± 1.90^ABc^	411.34 ± 3.40^Ad^	409.19 ± 1.80^Cd^	417.30 ± 9.00^Ae^	328.00 ± 6.40^BCa^	375.08 ± 8.30^Ab^
DPPH antioxidant activity (mg TE/L)	UV‐1	820.24 ± 54.20^Ab^	1010.56 ± 85.30^Ad^	843.33 ± 3.50^ABbc^	878.91 ± 35.90^Aa^	876.22 ± 32.60^BCbc^	887.22 ± 34.00^Ac^	957.32 ± 16.50^Cd^
	UV‐2	834.89 ± 54.10^Ab^	1002.95 ± 85.60^Ad^	859.36 ± 11.00^Cb^	895.92 ± 21.90^Aa^	887.31 ± 21.50^Cbc^	893.31 ± 9.00^Abc^	946.08 ± 15.10^ABcd^
	UV‐3	811.52 ± 32.10^Ab^	966.13 ± 61.70^Ae^	849.50 ± 26.20^Bbc^	816.08 ± 40.30^Aa^	877.45 ± 0.60^BCcd^	900.45 ± 33.10^Ad^	945.91 ± 12.70^Ae^
	UV‐4	784.93 ± 84.60^Ab^	1025.44 ± 85.60^Ae^	820.72 ± 32.00^Abc^	882.07 ± 22.40^Aa^	858.13 ± 1.20^ABcd^	867.13 ± 13.90^Acd^	917.53 ± 21.20^Cd^
	UV‐5	826.23 ± 48.00^Abc^	988.68 ± 90.20^Ae^	818.67 ± 45.30^Ab^	881.69 ± 55.50^Aa^	870.46 ± 3.50^BCbc^	884.46 ± 16.30^Ad^	937.50 ± 20.00^BCde^
	C	765.20 ± 47.90^Ab^	1031.80 ± 99.30^Bd^	869.23 ± 4.10^Cc^	898.70 ± 22.40^Aa^	864.71 ± 1.20^ABCc^	869.71 ± 8.20^Ac^	919.17 ± 6.40^Cc^
	HT	811.89 ± 70.60^Ab^	962.51 ± 61.00^ABc^	823.19 ± 38.90^Ab^	939.44 ± 30.70^Aa^	845.80 ± 24.40^Ab^	855.80 ± 38.60^Ab^	933.51 ± 14.20^BCc^
TAC (mg/L)	UV‐1	5.74 ± 0.10^BCcd^	4.33 ± 0.40^Aab^	5.58 ± 0.30^BCc^	3.60 ± 2.10^Aa^	5.39 ± 0.10^BCbc^	6.08 ± 0.10^Ccd^	6.90 ± 0.10^Ed^
	UV‐2	5.81 ± 0.10^Cdc^	4.36 ± 0.30^Aab^	5.64 ± 0.50^BCbc^	3.63 ± 2.40^Aa^	5.34 ± 0.20^Babc^	5.98 ± 0.10^BCc^	6.49 ± 0.10^Ad^
	UV‐3	5.58 ± 0.10^Aabc^	4.33 ± 0.20^Aa^	5.20 ± 0.10^Aab^	4.03 ± 2.10^Aa^	5.08 ± 0.20^Aab^	5.90 ± 0.30^ABbc^	6.71 ± 0.10^De^
	UV‐4	5.68 ± 0.10^ABbc^	4.33 ± 0.20^Aa^	5.43 ± 0.10^ABabc^	4.19 ± 2.00^Aab^	5.49 ± 0.10^Cabc^	6.00 ± 0.10^BCc^	6.41 ± 0.10^Bc^
	UV‐5	5.71 ± 0.20^BCabc^	4.62 ± 0.20^ABa^	5.49 ± 0.20^ABab^	3.86 ± 2.80^Aa^	5.37 ± 0.10^BCab^	6.55 ± 0.10^Dbc^	7.18 ± 0.10^Cc^
	C	5.87 ± 0.10^Dbcd^	4.74 ± 0.30^Bb^	5.91 ± 0.10^Cbcd^	3.16 ± 2.00^Aa^	5.69 ± 0.10^Dbc^	6.52 ± 0.10^Dcd^	7.05 ± 0.10^ABc^
	HT	5.82 ± 0.10^Cdcd^	4.35 ± 0.40^Aab^	5.51 ± 0.30^ABcd^	3.42 ± 1.90^Aa^	5.14 ± 0.20^Abc^	5.76 ± 0.10^Acd^	6.32 ± 0.20^Fc^

*Note*: Lowercase letters denote significant statistical differences within rows via the ANOVA Duncan test (*p* < .05); identical letters imply no statistical difference between samples (*p* ≥ .05). Uppercase letters indicate significant differences within columns as per the ANOVA Duncan test.

At the beginning of the storage, UV‐C and HT‐treated samples showed higher DPPH radical scavenging activities than C samples. This can be attributed to the enhanced extraction of antioxidant compounds in shalgam juice due to the effects of UV‐C and HT treatments.

On the day 0, DPPH antiradical activity of UV‐C‐treated samples increased by 2.58–7.98%, while HT‐treated sample showed 6.10% increase. However, there was no statistically significant difference in the initial DPPH values among UV1‐4, HT, and control samples (*p* < .05). Over the storage, all samples showed an increase in DPPH scavenging activity as compared to their initial values. The increase in antiradical activity was highest in the samples stored for 15 days. At the end of the 150‐days of the storage, all samples, except for UV‐4, showed higher antiradical activity than the control sample while The UV‐1 sample had the greatest ability. According to Alothman et al. (2009), UV‐C treatment could affect the extractability of antioxidants in fruit juice depending on various factors such as the delivered dose of UV‐C, exposure time, and the specific raw materials used. Therefore, it is likely crucial to carefully assess the UV‐C processing variables to prevent any potential negative impact on the quality of fruit based juices.

TAC results for C, HT, and UV‐C‐treated samples ranged from 3.16 to 7.18 mg/L, as shown in Table 5. Contrarily, TAC values for shalgam juice varied between 94 and 238 mg/L (with an average of 192 mg/L) by (Tanriseven et al., 2018). The observed variation could be attributed to the intricate interactions between acidity (caused by the microflora), the variations in the formulation, fermentation conditions and the extraction variables of phenolics (Tanriseven et al., 2020). On the storage days of 0, 15, 30, 90, 120 (excluding UV‐4), and 150 (excluding UV‐4), UV‐C‐treated samples exhibited lower TAC values as compared to the C‐sample. However, UV‐C‐treated samples showed higher TAC values than the C‐sample at 60^th^ day of the storage. On the other hand, HT sample exhibited lower TAC values as compared to the control sample throughout the storage period, except on the 60^th^ day (*p* < .05). Organic molecules absorb photons of UV light with a wavelength of 253.7 nm, which can impact conjugated bonds found in compounds like aromatic rings, double rings, and those containing disulfide bonds. As a result, this interaction may have the potential to decrease the anthocyanin content (Pala & Toklucu, 2011). Turker et al. (2004) reported that anthocyanins that are acylated with aromatic acids had greater stability than their nonacylated counterparts. Furthermore, it has been observed that the concentration of polymeric pigments increased with the increasing temperature and the extension of storage time. However, Turker et al. (2004) reported that pasteurization did not have any significant impact on TAC at any storage temperature.

### 3.4 Influence of in vitro simulated digestion on TPC, TAC, and DPPH radical scavenging activity

The in vitro bioaccessibility of TPC, TAC, and DPPH antioxidant activity for UV‐3 and HT samples is presented in Table 6. The decision to focus on UV‐3 and HT samples for in vitro bioaccessibility testing was based on several factors. First, the UV‐3 sample showed the highest counts of LAB and the lowest counts of TYM and TMAB, indicating better microbiological quality. This sample was compared with the HT sample, which represents the commercial application. The measured TPC values for UV‐3‐coded samples were 98.64 mg GAE/L in the oral phase, 205.64 mg GAE/L in the gastric phase, 183.84 mg GAE/L in the IN phase, and 288.73 mg GAE/L in the OUT phase following in vitro gastrointestinal digestion. On the other hand, for the HT‐treated beverages, the TPC values were measured as 77.46 mg GAE/L in the oral phase, 139.72 mg GAE/L in the gastric phase, 89.59 mg GAE/L in the IN phase, and 303.51 mg GAE/L in the OUT phase. The reduced levels of phenolic compounds in the oral phase as compared to the undigested sample could be attributed to the limited solubility of these compounds in saliva and the relatively shorterduration of this phase (Ucar & Karadag, 2019). The susceptibility of phenolic compounds to instability in the alkaline environment of the intestinal tract suggests that a significant portion of polyphenols was highly sensitive to neutral or slightly alkaline conditions (Quan et al., 2017). The highest TPC, TAC, and DPPH radical scavenging activity was observed during the gastric digestion phase. The increase in phenolic compounds observed under gastric conditions implies that the acidic pH and digestive enzymes may aid in the liberation of phenolics that are present in the shalgam. The UV‐3 samples consistently exhibited higher antioxidant/bioactive properties than the HT‐coded sample both before and during the digestion process. Furthermore, it was observed that the OUT values for TPC, TAC, and DPPH radical scavenging activities were higher than the IN values for all tested samples. This suggests that these compounds become less bioavailable in serum after intestinal digestion (Dogan et al., 2021). As well, the stability of anthocyanins is widely recognized to be higher under acidic pH and lower under alkaline pH conditions. Furthermore, the pH stability of anthocyanins could be influenced by their specific chemical structures (Slavu (Ursu) et al., 2020). Toktaş et al. (2018) studied the in vitro bioaccessibility of bioactive properties of shalgam during its fermentation and reported a significant decrease in the recovery, particularly for parameters including TPC, TAC, and antioxidant capacity, as a result of the in vitro digestion process. Overall, it could be concluded that UV treatment showed a positive effect on the in vitro bioaccessibility of TPC, TAC, and DPPH radical scavenging activities as compared to those of HT‐treated samples.

**TABLE 6:** Changes in TPC, TAC, and DPPH antioxidant activities of UV‐3‐ and HT‐coded samples depending on in vitro gastrointestinal digestion conditions.

	Shalgam beverage stored for 150 days	*In vitro* gastrointestinal digestion				BI (%)
		Mouth	Gastric	Small intestinal		
				IN	OUT	
UV‐3						
TPC (mg GAE/g)	481.89 ± 7.50^e^	98.64 ± 1.21^a^	205.64 ± 06.75^c^	183.84 ± 2.57^b^	288.73 ± 4.55^d^	38.90
TAC (mg/L)	6.71 ± 0.10^d^	1.16 ± 0.01^b^	3.56 ± 0.02^c^	1.03 ± 0.01^a^	7.46 ± 0.04^e^	12.13
DPPH (mg TE/L)	945.91 ± 12.70^e^	285.63 ± 1.03^b^	328.84 ± 3.15^c^	229.46 ± 2.52^a^	604.57 ± 5.31^d^	27.51
HT						
TPC (mg GAE/g)	375.08 ± 8.30^e^	77.46 ± 0.27^a^	139.72 ± 0.72^c^	89.59 ± 0.85^b^	303.51 ± 9.52^d^	22.79
TAC (mg/L)	6.32 ± 0.20^d^	0.73 ± 0.01^b^	2.52 ± 0.01^c^	0.46 ± 0.01^a^	6.36 ± 0.01^d^	6.74
DPPH (mg TE/L)	933.51 ± 14.20^e^	142.97 ± 0.69^b^	201.65 ± 1.97^c^	117.38 ± 0.01^a^	584.97 ± 0.02^d^	16.71

*Note*: Statistical differences in the same rows are expressed with lower case letters (*p* < .05). Statistical differences in the same rows are expressed with the same lower case letters (*p* < .05). Abbreviations: BI, Bioaccessibility index; DPPH, 2,2‐Diphenyl‐1‐picrylhydrazyl; TPC, Total phenolic content.

### 3.5 Sensory properties

The results of the sensory analysis of shalgam juices stored for 150 days are tabulated in Table 7. In terms of sensory parameters, UV‐treated samples received similar ratings to the C sample. However, HT‐treated sample received significantly lower sensory scores (*p* < .05). In other words, UV‐C‐treated shalgam samples were generally preferred more by the panelists than the HT‐treated one. No significant differences were observed between UV‐treated and control samples in terms of appearance, taste, aroma, consistency, and overall apprecation (*p* > .05). Consistent with Tangüler et al. (2020), the sensory evaluation results of this study also showed a decrease in sensory ratings, especially for cooked carrot and odor, when shalgam juices were treated with HT. However, Abdul Karim Shah et al. (2016) reported no significant difference between UV‐C‐treated juices and fresh juices. These findings showed that using UV‐treatment instead of conventional HT for shalgam beverages could provide better sensory quality to shalgam.

**TABLE 7:** Sensory properties of the shalgam juice samples.

Operation codes	Sensory parameters							
	Appearance	Taste	Odor	Aroma	Color	Turbidity	Consistency	Overall appreciation
UV‐1	8.40 ± 0.40^b^	8.20 ± 0.30^b^	7.80 ± 0.40 ^b^	8.30 ± 0.60^b^	8.40 ± 0.10^b^	7.80 ± 0.30^a^	7.90 ± 0.20^b^	8.10 ± 0.30^b^
UV‐2	8.60 ± 0.20^b^	8.20 ± 0.30^b^	8.30 ± 0.10^c^	8.40 ± 0.40^b^	8.50 ± 0.30^b^	7.90 ± 0.10^a^	8.20 ± 0.20^b^	8.30 ± 0.20^b^
UV‐3	8.70 ± 0.30^b^	8.40 ± 0.50^b^	8.40 ± 0.30^c^	8.40 ± 0.70^b^	8.50 ± 0.20^b^	8.20 ± 0.20^b^	8.10 ± 0.30^b^	8.40 ± 0.20^b^
UV‐4	8.60 ± 0.30^b^	7.90 ± 0.00^b^	8.20 ± 0.10^c^	8.30 ± 0.20^b^	8.50 ± 0.20^b^	7.90 ± 0.30^b^	8.10 ± 0.20^b^	8.20 ± 0.30^b^
UV‐5	8.40 ± 0.20^b^	8.20 ± 0.30^b^	7.80 ± 0.40^b^	7.90 ± 0.20^b^	8.70 ± 0.10^b^	8.00 ± 0.30^b^	8.20 ± 0.20^b^	8.20 ± 0.20^b^
C	8.60 ± 0.30^b^	8.10 ± 0.60^b^	7.80 ± 0.00^b^	8.40 ± 0.10^b^	8.40 ± 0.10^b^	8.10 ± 0.30^b^	8.10 ± 0.20^b^	8.10 ± 0.20^b^
HT	7.60 ± 0.20^a^	5.70 ± 0.30^a^	5.90 ± 0.40^a^	6.00 ± 0.30^a^	7.20 ± 0.30^a^	7.60 ± 0.20^a^	7.50 ± 0.10^a^	6.60 ± 0.50^a^

*Note*:Statistical differences in the same rows are expressed with the same lower case letters (*p* < .05).

## 4 CONCLUSION

In the present study, the effects of UV‐C and HT on the physicochemical, microbiological, bioactive characteristics, in vitro bioaccessibility of bioactive properties, and sensory attributes of shalgam beverages were extensively evaluated and compared with non‐treated samples. The results showed that the samples generally met the desired pH range (except on 90^th^ and 120^th^ days) and total acidity standards (except UV‐3, UV‐5, and HT samples on the 30^th^ day) according to TSE‐standard requirements. Moreover, all samples exhibited lower L* and higher *ΔC* values on the 0^th^ day as compared to the 150^th^ day. Storage duration and treatment significantly influenced the microbiological properties of the samples, with UV‐treatment generally yielding better microbiological quality. The bioactive properties of samples were affected from both storage time and the processing method applied. At the end of the storage period, UV‐treated samples showed higher DPPH activity, TPC, and TAC as compared to both control and HT samples. DPPH radical scavenging activity, TPC, and TAC were unstable throughout the in vitro digestion process. Overall, this study showed that UV‐C treatment could be used as an alternative to HT, providing the desired sensory, microbiological, and bioactive characteristics in shalgam juice production.

## AUTHOR CONTRIBUTIONS

**Kubra Feyza Erol:** Formal analysis (equal); Investigation (lead); Methodology (equal); Writing – original draft (equal), Visualisation. **Gozde Kutlu:** Investigation; Data curation (equal); Writing – original draft (equal), Visualisation, Review & editing. **Mehmet Baslar:** Conceptualization (equal); Methodology (equal); Resources (lead); Supervision (equal). **Fatih Tornuk:** Conceptualization (equal); Supervision (equal); Writing – original draft (supporting).

## FUNDING INFORMATION

This research was funded by the Scientific and Technological Research Council of Türkiye (TUBITAK) under Grant No: ARDEB 214O417. Moreover, the authors would like to thank TUBITAK for generously covering the open‐access publication costs.

## CONFLICT OF INTEREST STATEMENT

The authors state that they have no conflicts of interest.

## Data Availability

The data that support the findings of this study are available on request from the corresponding author.
